# PointCloud-At: Point Cloud Convolutional Neural Networks with Attention for 3D Data Processing

**DOI:** 10.3390/s24196446

**Published:** 2024-10-05

**Authors:** Saidu Umar, Aboozar Taherkhani

**Affiliations:** School of Computer Science and Informatics, De Montfort University, Leicester LE1 9BH, UK; saeedumar5@gmail.com

**Keywords:** deep learning, point cloud data, attention mechanism, 3D data

## Abstract

The rapid growth in technologies for 3D sensors has made point cloud data increasingly available in different applications such as autonomous driving, robotics, and virtual and augmented reality. This raises a growing need for deep learning methods to process the data. Point clouds are difficult to be used directly as inputs in several deep learning techniques. The difficulty is raised by the unstructured and unordered nature of the point cloud data. So, machine learning models built for images or videos cannot be used directly on point cloud data. Although the research in the field of point clouds has gained high attention and different methods have been developed over the decade, very few research works directly with point cloud data, and most of them convert the point cloud data into 2D images or voxels by performing some pre-processing that causes information loss. Methods that directly work on point clouds are in the early stage and this affects the performance and accuracy of the models. Advanced techniques in classical convolutional neural networks, such as the attention mechanism, need to be transferred to the methods directly working with point clouds. In this research, an attention mechanism is proposed to be added to deep convolutional neural networks that process point clouds directly. The attention module was proposed based on specific pooling operations which are designed to be applied directly to point clouds to extract vital information from the point clouds. Segmentation of the ShapeNet dataset was performed to evaluate the method. The mean intersection over union (mIoU) score of the proposed framework was increased after applying the attention method compared to a base state-of-the-art framework that does not have the attention mechanism.

## 1. Introduction

Point cloud processing using deep learning methods has gained a lot of attention. Point clouds are a set of data points in space to display 3D geometry. They have gained popularity and wide usage in several domains. The rapid growth in 3D technologies and 3D sensors has made point cloud data increasingly available [1]. Additionally, the world is in three dimensions, thus point clouds are a suitable format for representing the real world in XYZ coordinates. The usage of point clouds extends to a variety of disciplines and the 3D nature of point clouds makes them the appropriate format for autonomous driving, robotics, virtual and augmented reality, heritage preservation, and many more applications [2]. The availability of point cloud data has raised a need for advanced deep learning methods to process 3D point clouds.

Deep learning techniques have been used to perform different kinds of processing on image and video data [3,4,5,6]. Some deep learning techniques that have been applied to point clouds include classification, detection and tracking, reconstruction, and segmentation [7]. For over 50 years, the segmentation of images has been the focus of several researchers. The purpose of segmentation is to break an image into subregions with similar features, and it has had a great impact on computer vision [7,8]. Point cloud segmentation has faced challenges due to the unstructured nature of the point cloud data and its highly redundant and uneven nature [9]. Although various point cloud segmentation methods exist, designing deep learning techniques for this purpose remains a challenging task. Developing advanced methods in this field will improve the accuracy of computer vision in 3D space for different applications.

Attention mechanisms have had a great impact on deep learning by improving the accuracy of models and their performance [10]. Attention mechanisms help the models concentrate on the most important features of input data [11]. There are several types of attention mechanisms. One of the early applications of attention mechanisms in point cloud data is the shuffle attention model [12]. A little research has been carried out on improving the performance of point cloud convolutional neural networks (CNNs) with attention mechanisms. Unlike pixel images, there have been few usages of attention mechanisms directly applied to point cloud data [7]. The unstructured and unordered nature of point cloud data causes each point to have specific importance, and using a processing approach that evenly processes the points is not suitable.

Therefore, in this research, an attention mechanism is proposed for processing point cloud data. The attention mechanism is directly applied to point cloud data without mapping them to a continuous space. It learns the importance of points using training data and places specific emphasis on each point. The proposed attention mechanism is added to a network called ConvPoint [9]. The performance of the proposed method was compared with the base method without the attention mechanism, i.e., ConvPoint [9]. Additionally, the method was compared with other state-of-the-art methods.

The structure of this paper is as follows. Section 2 reviews the existing literature on deep learning methods for point clouds and attention mechanisms. Section 3 explores the proposed method and its components. Section 4 discusses experiments and results. Finally, Section 5 concludes the paper.

## 2. Literature Review

For different applications, such as perception and localisation, which are key for navigation in autonomous vehicles, visual data processing plays an important role [13]. Image data extracted from a camera are usually represented in 2D; however, the 2D data lack the required geometric and volumetric information [13]. A point cloud is a representation tool for 3D data. In this section, initially, deep learning methods for point clouds are reviewed. Then, attention mechanisms in deep learning for images and point clouds are discussed.

### 2.1. Deep Learning Methods for Point Clouds

A key challenge in point cloud processing is the inefficiency of traditional CNNs in processing the original form of point cloud data [14]. The sparse, unstructured, and unordered nature of point clouds makes the standard CNN architecture less effective. To address these issues, refs. [15,16] proposed the pre-processing of point cloud data into voxel representations or 2D images. Three-dimensional point clouds can be converted into 2D images using multi-view-based methods or into a 3D volumetric representation to be processed by well-known 2D or 3D convolutional networks [7]. Although these methods might ease the implementation, the loss of certain geometric information of the point cloud data is the cost of this implementation. This significant gap represents a critical challenge in the field and identifies a huge demand for approaches that can directly process point cloud data without compromising its intrinsic characteristics. Our proposed method, which is a point-based approach, addresses this limitation by leveraging the power of deep learning to develop an architecture that preserves the original structure of the point cloud data, thus maintaining important geometric and spatial information. The pointwise method does not use any voxelization or other projection methods.

There are different types of pointwise methods. Pointwise MLP methods process each point independently using shared multilayer perceptrons (MLPs). Then, the output features are aggregated (global aggregation) using an aggregation function. PointNet [17] is a pioneering pointwise MLP method that uses max pooling for global feature aggregation. It was improved by introducing a structure to extract global and local features to create PointNet++ [14]. PointNet++ uses a max pooling method for local feature aggregation. The dynamic graph CNN (DGCNN) [15] was proposed to aggregate local region information using the feature of a centre point and the differences between the feature of the centre point and the features of its k nearest neighbour points. DGCNN only considers the pair relation for the centre point. PointWeb [16], a pointwise MLP method, was proposed to consider all pairs of points in a local region using a module called adaptive feature adjustment (AFA). One of the challenges of the pointwise MLP methods is their high computational cost.

Convolution-based methods for 3D point clouds are another group of deep neural networks that use specific convolution kernels to process 3D point clouds. Three-dimensional discrete convolution methods are an important group of convolution-based methods, and they use convolutional kernels on regular grids and the offset of each point to a centre point to define weights for neighbouring points. For instance, Hua et al. [18] used uniform grids to define convolutional kernels. They transformed 3D point clouds into uniform grids and assigned the same weight to the points falling in the same cell or subdomain. The mean value of the features of the points in a cell is multiplied by its corresponding kernel weight and summed with the other weighted mean values on all other cells in the kernel domain to calculate the output. In the spherical convolutional kernel proposed by Lei et al. [19], multiple volumetric bins were created by partitioning a 3D spherical neighbouring region. A learnable weighting matrix was assigned for each bin.

Three-dimensional continuous convolution methods are another group of convolution-based methods for processing 3D point clouds. Despite the 3D discrete convolution methods that consider discrete regions or domains, they used convolutional kernels on a continuous space. The weights in the convolutional kernel for neighbouring points are related to the continuous spatial distance from a centre point. For instance, RS-Conv [20] uses an MLP to implement a convolution. The MLP is trained to map low-level relations between input points such as Euclidean distance and the relative position to high-level relations between points in the local subset. Then, the output of the MLP is used to calculate the weighted sum over the given subset. ConvPoint [9] is another method that performs convolution in two parts, namely the spatial and feature parts. The locations of the kernel points are selected randomly from a unit sphere. The kernel points and the position of input points are applied to an MLP to create kernel weights. The convolutional layer can be used as a building block of complex networks. Different structures in the classical neural networks can be used to design new network structures for point cloud processing. In this project, an attention mechanism is used with a 3D continuous convolution method to design a new network structure.

Wang et al. [8] addressed one of the critical limitations, the inability of discrete CNNs to handle the unstructured nature of point cloud data, thus proposing a framework that generalises discrete CNNs to deal with point clouds. While [8] made progress in adapting CNNs to process point cloud data, the framework faces challenges in handling large-scale, real-time point cloud data, which is critical for many practical applications. The performance of this framework in scenarios with varying point densities or occlusions remains unclear. Our work is expected to build upon this framework to reduce some of the limitations and to improve point cloud processing in real-world applications and dynamic environments.

### 2.2. Attention Mechanisms in Deep Learning Methods for Images

Attention mechanisms have improved image segmentation significantly; however, their applications in point cloud data have been limited. There have been promising applications of attention mechanisms in 2D image processing. Approaches applied to 2D images include spatial channel attention [10], shuffle attention [11], and the convolution block attention module [21]. These approaches have improved model performance by effectively capturing channel dependencies and pixel-level relationships.

Using image-based attention mechanisms directly on 3D point cloud data has been faced with challenges. Often, preprocessing or data conversion is required, leading to potential data loss. Additionally, the unique spatial structure of the 3D point cloud data may not be fully leveraged.

Our work aims to bridge this gap by developing attention mechanisms designed to work on 3D point cloud data. This framework will address the challenges caused by the unstructured nature of point cloud data while maintaining the advantages of attention mechanisms.

### 2.3. Attention Mechanisms in Deep Learning for Point Clouds

Yang et al. [22] proposed the attention-based point network (AttPNet), a network that utilises attention mechanisms to perform channel weighting and global feature masking on feature areas. AttPNet has two branches, where one branch deduces global features from point sets using convolutional layers to create a channel attention block focusing on the key channels of the data. The other branch performs the calculation of an attention mask for every point. Subsequently, the authors designed a point cloud dataset of electron cryo-tomography (ECT) and used these data to show the AttPnet’s capacity of handling fine-grained structures. The authors aimed to design a model that handles fine-grained structures. The attention mechanisms use the MLP. Additionally, they only use the features, and the exact position of the points was not applied to the MLP. In our research, a point cloud convolutional layer that accepts the position of the input points in addition to the input features is used to design an attention mechanism.

Hu et al. [23] introduced an attention-based module for extracting local features in their semantic framework for point cloud data labelling. Although this design achieved a modest output, it had limitations in fully utilising geometric calculations of neighbouring points. Deng and Dong [24] designed a global attention network for point cloud segmentation to address the problem of learning long-range dependencies from 3D point clouds, which has been a challenging problem in the processing of 3D point clouds. The global attention network, or GA-Net, comprises a global attention module that is point-independent and another global attention module that is point-dependent for gathering background information on 3D points. Both [25,26] made significant progress in utilising geometric calculations of neighbouring points and learning long-range dependencies but face limitations in balancing computational complexity with performance.

Several researchers focus on spatial encodings whilst ignoring the channel relationships, making feature learning insufficient. Hence, the lightweight attention module (LAM) was developed in [27] to improve the performance while adopting a new convolutional function and introducing a channel-based attention mechanism. However, its integration with existing networks may not fully exploit the unique properties of point cloud data.

One of the issues with working with point clouds is the inability to fully utilise the geometric information of neighbouring points. This prompted Feng et al. [28] to propose and design the local attention–edge convolution (LEA-Conv) layer. This layer is an extension of the works proposed in [14,15,29]. The LAE-Conv model builds a graph of neighbourhood points along several routes. Consequently, a search strategy was proposed in [28] to use a multidirectional search to find all points in the neighbourhood across 16 directions systematically within a ball query to generalise the local geometric shape over the space. Additionally, a pointwise spatial attention block was proposed to capture information in the spatial dimension. The output features of the LEA-Conv layer were applied to the spatial attention block to create outputs that capture the spatial dependency of the points. The spatial attention block does not consider the correlation in the different channels. In this research, a channel attention mechanism is proposed.

This review highlighted the importance of attention mechanisms on model performance. A critical gap exists in designing attention mechanisms that work directly on the geometric information contained in 3D point cloud data. These attention mechanisms will significantly improve the accuracy of existing 3D point CNNs.

## 3. Methodology and Framework

This section covers a brief description of ConvPoint [9], which is used as the base of the proposed method after a discussion of the problem statement. The point cloud convolutional layer in [9] is used as a base method in this research because it is a convolution method that directly processes point clouds. It does not have an attention mechanism. Moreover, its output is also point clouds. Therefore, its output has an acceptable format that can be applied to the proposed attention mechanism in this paper, which is designed to directly work on point cloud data. The proposed attention mechanism will be introduced in Section 3.3. Lastly, the final network structure will be discussed.

### 3.1. Problem Statement

In this research, a method is proposed to enhance the performance of ConvPoint [9] with a spatial attention module that is inspired by the convolutional block attention module (CBAM) [21]. ConvPoint is an oversimplification of a discrete convolutional neural network [9]. The CBAM is an attention module for the usual convolutional neural networks used for normal data such as images [21]. The structure of point cloud data is different from the usual data produced by a classical convolutional layer and consequently, it needs a new attention mechanism. In this research, we design a channel attention mechanism to boost the performance of ConvPoint [9].

Suppose a convolutional layer works directly on point clouds. The layer has a kernel function and an input in the form of point clouds. In the convolutional layer for point clouds, the following kernel *K* and input *P* that have compatible dimensionality are used: $K=\left\{{\left(c_{i},w_{i}\right),c_{i}\in R^{3},w}_{i}\in R^{n},i\in ⟦1,\left|K\right|⟧\right\}$ and $P=\left\{\left(p,x\right)\right\}=\left\{{(p_{i},x}_{i}),p_{i}\in R^{3},x_{i}\in R^{n},i\in ⟦1,\left|P\right|⟧\right\}$, where $\left|K\right|$ is the number of elements in the kernel and $\left|P\right|$ shows the size of the input set, i.e., the number of points that are in the input set P. The convolutional layer for point clouds accepts point cloud data composed of several input points and corresponding features, i.e., $P=\left\{\left(p,x\right)\right\}$, where x is an n-dimensional feature from the input feature space corresponding to an input point, i.e., p in the 3D space. The convolutional layer uses the kernel *K* to create an output composed of the place of the output points, i.e., q, and their features (y), i.e., $Q=\left\{\left(q,y\right)\right\}=\left\{{(q_{i},y}_{i}),q_{i}\in R^{3},y_{i}\in R^{n},i\in ⟦1,\left|Q\right|⟧\right\}$.

### 3.2. Continuous Convolutions for Point Cloud Processing (ConvPoint)

A continuous convolutional layer was proposed in [9] by adjusting the discrete convolutional layer used for the common 2D image datasets to process point cloud data. The following two operations are performed in ConvPoint [9] to map the input P to the output Q: 1—point selection, and 2—convolution on point sets.

(a) Point selection.

Each point q in the output set, $Q=\left\{\left(q,y\right)\right\}$, is selected randomly from input points that are in $P=\left\{\left(p,x\right)\right\}$ using the random method in [9]. A score is allocated to each input point and whenever a point is selected randomly, its score is increased by 100. Additionally, the scores of its neighbour points are increased by 1. Increasing the scores of a selected point and its neighbour points reduces their chance of being selected in the next selection procedure. This method is used to give a chance to all points to be selected and to reduce the probability of selecting repeated points. The selection procedure is continued until the required output points are selected.

(b) Convolution on point sets.

After selecting the output points, for each output point q, the *k*-*d* tree method [9] is used to find local neighbour points in $P=\left\{\left(p,x\right)\right\}$ to create a subset of points for each q. Then, point convolution is applied to the subset of points using (1).

(1) $$y=\beta +\frac{1}{\left|P\right|}\sum_{j=1}^{\left|P\right|}\sum_{i=1}^{\left|k\right|}w_{i}x_{j}f\left(p_{j}-c\right)$$

where β is a bias parameter, $\frac{1}{\left|P\right|}$ is used to reflect the input set size to have robustness against input size variation, and $f\left(.\right)$ is a geometrical weighting function to distribute the input $P=\left\{\left(p,x\right)\right\}$ onto the kernel $K=\left\{\left(c,w\right)\right\}$. It accepts the relative distance between each input point p and all the kernel elements $\left\{c\right\}$, i.e., $p_{j}-c$, to create a weight in R corresponding to an input point as shown in (2).

(2) $$f:R^{3}\times {\left(R^{3}\right)}^{\left|K\right|}\to R$$

A simple neural network, i.e., the multilayer perceptron (MLP), is trained to act as $f\left(.\right)$. The MLP is used to build the general function $f\left(.\right)$, as this approach is easier than building the general function from scratch. For each kernel in the convolution operation, spatial and feature parts were processed independently. The parameters were optimised using gradient descent during training.

The locations of the kernel elements $\left\{c\right\}$ are initialised by randomly selecting them from the unit sphere. Training parameters of the convolutional layer, i.e., $\left\{c\right\}$ and $\left\{w\right\}$, and the training parameters of the MLP are optimised using the gradient descent method.

### 3.3. The Proposed Attention Mechanism for Point Cloud Continuous Convolutional Layer

In this research, the attention module proposed in [21] for the common convolutional layer inspired us to design an attention mechanism for the point cloud convolutional neural network described in Section 3.2. A channel attention mechanism for the continuous convolutional layer for point clouds (ConvPoint) is proposed in this research. The proposed attention mechanism is designed to perform different operations such as average pooling and max pooling operations on point cloud data extracted from a ConvPoint layer.

In the proposed attention mechanism, an input feature map is converted into a channel attention map using the proposed method. The process of channel attention is given by (3).

(3) $$F^{\prime }=M_{c}\left(P\right)⨂P$$

where $P=\left\{\left(p,x\right)\right\}$ is the input to the attention block, which is produced from its previous ConvPoint layer, ⨂ denotes multiplication of the elements, and $M_{c}\left(.\right)$ is a channel attention mechanism. As the input of ConvPoint is composed of two parts, i.e., p and x, in this paper, a mechanism was proposed to deal with the two parts of the attention mechanism.

**Figure 1:**
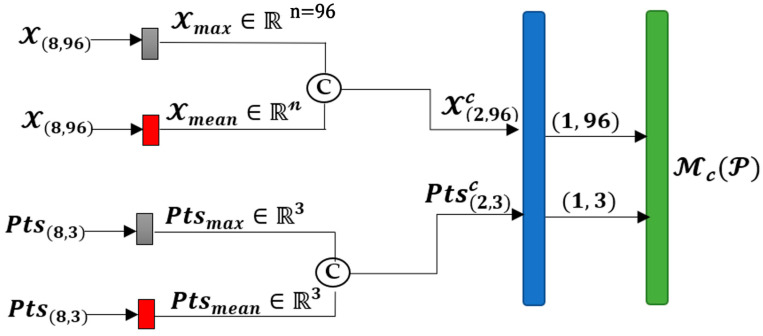
The structure of the proposed channel attention mechanism for the point cloud convolutional layer. The numbers in the parentheses show the size of each tensor before and after each operation. In this sample network, |*P*| = 8 and *n* = 96. The grey, red, blue, and green boxes represent max pooling, average pooling, the ConvPoint layer, and sigmoid operations, respectively. The C stands for concatenation.

First, pooling operations, i.e., max pooling and average pooling, were applied to the features $X=\left[x_{1}x_{2}\ldots x_{\left|p\right|}\right]$, where $x_{i}\in R^{n}\;\mathrm{for}$ $i\in ⟦1,\left|P\right|⟧$, $X_{max}\in R^{n}$, and $X_{mean}\in R^{n}$ are created after the max pooling and average pooling on the features related to the $\left|P\right|$ input points. Then, the results are concatenated as shown in Figure 1 using (4).

(4) $$X^{c}={C(X}_{max},X_{mean})$$

where $C\left(.\right)$ is the concatenation function combining the two inputs with a size of $\left(1,n\right)$ to create a concatenated output with a size of $\left(2,n\right)$.

In the next step, we propose to perform pooling on the points in the input points, i.e., $Pts=\left[p_{1}p_{2}\ldots p_{\left|P\right|}\right]$*,* where $p_{i}\in R^{3}\;\mathrm{for}$ $i\in ⟦1,\left|P\right|⟧$ to create two points in the 3D space corresponding to the two features extracted from max and average pooling. The results for the two operations on Pts are ${Pts}_{max}\in R^{3}$ and ${Pts}_{mean}\in R^{3}$. The two vectors are concatenated using (5).

(5) $${Pts}^{c}={C(Pts}_{max},{Pts}_{mean})$$

${Pts}^{c}$ has an appropriate size of $\left(2,3\right)$ to be combined with $X^{c}$ to create an input $P^{c}$ to be applied to a ConvPoint layer, as shown in Figure 1. After passing the pairs of ${Pts}^{c}$ and $X^{c}$ to the Conv layer, the output will be passed to a σ function using (6).

(6) $$M_{c}\left(P\right)=\sigma \left(ConvPoint\left({Pts}^{c},X^{c}\right)\right)$$

The output of the attention block, i.e., $M_{c}\left(P\right)$, is obtained by multiplying by the original input P using the element-wise multiplication in (3) and the results $F^{\prime }$ will be added to the original input.

### 3.4. The Network Structure

**Figure 2:**
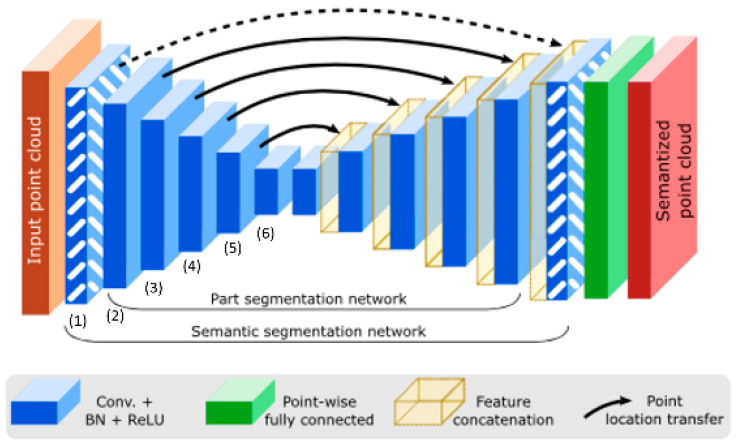
Semantic segmentation network graphical display, as fetched from [9].

The network used in this project has a structure similar to U-Net, used in [9] for segmentation. The original network without the attention mechanism is given in Figure 2. The network is composed of two main parts, an encoder and a decoder. There are six ConvPoint layers in the encoder. Each ConvPoint layer is a part of six blocks, demonstrated in Figure 2, given by a number from (1) to (6). The output of each ConvPoint layer comprises point cloud data and it has two parts, i.e., the position of the points ${Pts}_{i}$ and the corresponding features, i.e., $X_{i}$. The proposed attention module is applied to the network in different parts to find the best locations for the attention mechanism. For instance, it is applied after the final layer in the encoder just before entering the decoder, i.e., ${Pts}_{6}$ and $X_{6}$. The results were demonstrated in Section 4. The point cloud convolutional neural network with the proposed attention is called PointCloud-At.

Since input point clouds are given in different sizes, 2500 points are selected randomly, and the label of every point in the input is determined as output for segmentation. Cross-entropy loss is calculated for each point and the scores at the shape level are calculated accordingly. The scores for the network are the instance average intersection over union (mIoU) [9].

## 4. Experiments and Results

Different experiments were carried out on the ShapeNet dataset [30] to thoroughly evaluate the proposed channel attention mechanism on point cloud data. These and the results are discussed in this section. The dataset is also described in Section 4.1.

### 4.1. Dataset

The proposed attention mechanism is evaluated on the ShapeNet dataset [30], which contains point clouds. Different experiments were run to evaluate the attention mechanisms from different perspectives of the ShapeNet dataset [30]. The ShapeNet dataset is a rich annotated 3D representation of shapes. It provides semantic annotations. The ShapeNet dataset contains 16,680 models that belong to 16 shape categories. These categories are split into training and testing sets, with each category annotated with two- to six-part labels. Thus, it has around fifty (50) classes [9].

### 4.2. Experiments

To evaluate the proposed attention module, we modified the attention module by positioning it in several areas in the network, including the skip connections. The results were compared with the results of the base method described in Section 4.3.

In the first experiment for the proposed method, the proposed attention mechanism is applied between the encoder and decoder of the network, which is a U-net. The proposed attention module shown in Figure 1 is applied on the final layer of the encoder, i.e., ${Pts}_{6}$ and $X_{6}$. The matrix shape of the output of each operation is shown in Figure 1. After completing these operations, the training was run for 10 epochs. There is an improvement in the mean intersection over union (mIoU) score compared to the score of the original network without the proposed attention module.

In the next step, we applied the attention module on (${Pts}_{6}$, $X_{6}$) and (${Pts}_{5}$, $X_{5}$), i.e., the outputs of blocks 6 and 5 in Figure 2. The improvement in this was higher compared to the previous experiment, hence making it a better technique. Results are shown in the following sections. The best model was found, and it was trained for 200 epochs to be compared with the base method that was trained for the same number of epochs.

### 4.3. The Results of the Base Method

Initially, the base network in [9] was evaluated before applying the proposed attention module. The base method, i.e., ConvPoint, has shown a competitive performance compared to state-of-the-art methods [9] when it trains for 200 epochs. The framework is ranked amongst the best five frameworks (ranked number four) for both mean class intersection over union and mean intersection over union (mcIoU and mIoU) on the ShapeNet dataset [9]. We initially trained the base method for ten epochs using the initial set, and the final mean intersection over union (mIoU) for all shapes was 80.3% (Table 1).

### 4.4. The Results of the Proposed Method

The proposed point cloud convolutional neural network with attention, called the PointCloud-At method, was trained for ten epochs like the base method for a fair comparison. A low number of training epochs was used to reduce computation time while testing different situations to find appropriate hyperparameters/structures.

In the first experiment, we applied attention to (${Pts}_{6}$, $X_{6}$) and a score of 80.5% for the mIoU on all shapes was achieved. Secondly, we applied the attention mechanism to (${Pts}_{5}$, $X_{5}$), which gave a mIoU of 80.4%. In the next experiment, the proposed attention module was applied to (${Pts}_{4}$, $X_{4}$), and a mIoU score of 80.39% was achieved. Although different experiments showed improvement in the results compared to the base model, different combinations are tested to obtain a better score. Hence, the attention module is applied to the following outputs of the ConvPoint layers: (${Pts}_{6}$, $X_{6}$) and (${Pts}_{5}$, $X_{5}$). The mean intersection over union (mIoU) score of 81.45% was achieved, which is a better improvement compared to the previous cases. The results are given in Table 1.

In the next experiment, the attention mechanism was applied to the escape connection that connects the output of the fifth ConvPoint layer in the encoder to the corresponding layer in the decoder (Figure 2), i.e., (${Ptse}_{5}$, ${Xe}_{5}$). A mean intersection over union score of 80.30% was obtained, which is close to the score of the base method, as shown in the last row of Table 1. The simulation results in Table 1 show that attention modules applied to $\left({Pts}_{6},X_{6}\right)\;and\left({Pts}_{5},X_{5}\right)$ simultaneously could reach the best results. In the next experiments, the proposed method with the best results was trained for 200 epochs and compared to the base method trained for the same number of epochs (Table 2). Additionally, we explored using median pooling instead of max pooling, and the results are reported in the last row of Table 2.

In our research, we initially used max pooling in our attention mechanism; this is a common practise in many deep learning and attention mechanism architectures. We thought that different pooling operations might capture different aspects of the point cloud; hence, we explored median pooling. Unlike max pooling, which selects the maximum value, median pooling measures and selects the middle value. Using both max and median pooling resulted in the same score, showing that they both can perform on the same level in this case, providing flexibility on which operation to use. Obtaining the same score further indicates the robustness of our attention mechanism and its stability.

### 4.5. Comparison with State-of-the-Art Frameworks

Having carefully studied the quantitative results of various frameworks, as reported in [31], ConvPoint [9] has shown a comparative performance compared to the state-of-the-art methods. In this paper, an attention mechanism was proposed to improve the performance of ConvPoint [9]. The comparison of the proposed method with the other state-of-the-art methods is shown in Table 3. The results in Table 3 show that the proposed method in this research equates to several frameworks in some cases or outperforms them in other cases.

In this research, an attention mechanism was proposed to be added to existing CNNs for point clouds. The proposed attention mechanism was integrated into a recent method called ConvPoint [9], enhancing the method’s ability to focus on points containing important information. Consequently, the proposed method increased the mIoU score of ConvPoint [9] from 83.2 to 84.2.

Seventeen SotA methods are compared with the proposed method in Table 3. The proposed method has an accuracy higher than 15 SotA methods. While the base method, i.e., ConvPoint [9], has a mIoU score lower than SubSparseCN [32] and SPLATNet [33], applying the proposed attention method increased the accuracy of the proposed method to a value higher than the accuracies of SubSparseCN [32] and SPLATNet [33] that shows the effectiveness of the proposed method. The result in Table 3 shows the importance of the attention mechanism in deep neural networks and that applying the attention mechanism method to the other SotA methods can improve their accuracy.

**Table 3:** Comparison of the proposed method, i.e., PointCloud-At, with state-of-the-art methods.

Network	mIoU Score
PointCloud-At	84.2
SyncSpecCNN [34]	82.0
Pd-Network [35]	82.7
3DmFV-Net [36]	81.0
PointNet [17]	80.4
PointNet++ [14]	81.9
SubSparseCN [32]	83.3
SPLATNet [33]	83.7
SpiderCNN [37]	81.7
SO-Net [25]	81.0
PCNN [38]	81.8
KCNet [26]	82.2
RSNet [39]	81.4
DGCNN [15]	82.3
SGPN [40]	82.8
PointCNN [41]	84.6
KPConv [42]	85.1
ConvPoint [9]	83.2

Although KPConv [42] achieves the highest accuracy, it has a higher computational cost compared to the proposed method. The model size of KPConv was reported in [42]. The report shows that KPConv has 14.2 M parameters. However, the number of parameters in the proposed model is 1.3 M, which is much lower than the number of parameters in KPConv. The result shows that the number of parameters in the proposed model is about 11 times less than that of KPConv. The lower number of parameters in the proposed method makes it suitable for edge devices with limited computational resources and memory. Additionally, the low number of parameters reduces energy consumption. The results are shown in Table 4.

The proposed method uses a simple and efficient convolutional layer tailored for point clouds, and it is lightweight. It uses one kernel weight for each kernel point. However, KPConv [42] has a complex kernel filter, and each kernel point has a set of weights. Consequently, KPConv has a higher number of parameters compared to the proposed method.

## 5. A Discussion of the Applications of Point Cloud Processing

There is a growing demand for point cloud data processing in various applications that depend on 3D sensor data. Point cloud processing is a powerful tool with a wide range of applications across different scenarios in autonomous driving, robotics, virtual and augmented reality, medical imaging, digital surface modelling, automated building extraction, urban planning and visualisation, geographic information systems (GISs), and 3D modelling. The classification of dense point cloud data has been critical in creating detailed 3D models that have improved urban planning and development, medical imaging, autonomous driving, and many other fields.

Kurdi et al. [43] have used light detection and ranging (LiDAR) sensors for remote sensing applications. They demonstrated the potential of point cloud processing in urban planning. The authors proposed a method for automatic building point cloud filtering. The method divides building point clouds into different zones and extracts high tree crowns obstructing building structures. This application highlights the importance of processing point clouds extracted from 3D sensors for solving complex issues faced in urban planning, environmental management, and disaster management.

Maltezos et al. [44] explored point cloud processing in identifying several urban features. This work focused on improving the performance of building classification and extraction from densely populated areas, highlighting the technology’s ability to handle complex urban environments. Furthermore, ref. [45] discussed how the automation of extracting buildings from LiDAR data streamlines the creation of digital surface models (DSMs). Their work emphasises that such automation is vital for a range of applications, from smart city development to cartographic analysis, and it shows the wide impact of point cloud processing in urban planning and geospatial intelligence.

These applications collectively highlight the importance of point cloud processing in modern life. For instance, by providing detailed and accurate 3D representations of complex environments, this technology supports more informed decision-making in infrastructure management and urban planning, paving the way for smarter and more efficient cities in the future.

## 6. Conclusions, Limitations, and Future Work

This research proposed a deep point convolutional neural network for point cloud data using an attention mechanism. The study used an attention mechanism designed for point clouds to improve the performance of the network. The attention mechanism works using a channel attention module. The channel attention module was proposed specifically for point cloud data with inspiration from the CBAM [21] which is for ordinary convolutional layers acting on regular matrices (2D images). The proposed method overcomes the difficulties in processing scattered point cloud data compared to usual image or voxel data, which have regular shapes. In the proposed attention method, average pooling and max pooling are performed on the points in 3D space to focus on the informative parts of the data. Through several experiments and evaluations, we have shown that our proposed method enhances the performance of the base framework.

In this research, we designed a channel attention mechanism to boost the performance of ConvPoint [9]. The proposed method uses a max pooling operation on both the features and the positions of its input points in the 3D space. Additionally, it uses average pooling on the features and the position of its input points. The two operations create two points in the 3D space with their corresponding features. Then, the two points are applied to a ConvPoint [9] layer to create the outputs of the attention block. The ConvPoint [9] layer is a convolutional layer that is designed to operate on point clouds. Therefore, the proposed attention mechanism not only has a specific pooling operation on the input points (features and location of the inputs) but also contains a ConvPoint [9] layer that extracts appropriate outputs for the attention mechanism to be multiplied by the original input. Note that the proposed attention mechanism has learning parameters in the ConvPoint [9] layer, and they are adjusted during training to create a reliable attention mechanism using the training data. The proposed attention module is applied to the U-Net in different parts. These unique properties make this method different from other attention mechanisms, such as the method proposed in [28].

In [28], a convolutional layer called LAE-Conv layer was proposed to apply to point clouds. Whereas the ConvPoint [9] layer was used in our approach as a base method, in [28], a pointwise spatial attention module was proposed to capture the global dependencies. They used MLP layers in the attention block. Only features of the points are applied to the MLP and the positions of the points were not used. However, in our method, a new channel attention mechanism was proposed to put appropriate weights on the channel of the input point clouds. Additionally, it uses a ConvPoint [9] layer (instead of an MLP) inside the attention block that considers the position of the input points in addition to their features. The proposed attention block is designed in such a way that can be used with different convolutional layers to determine an output that corresponds to the importance of each channel.

In this project, ConvPoint [9] was used as the base method. ConvPoint [9] is an end-to-end deep neural network for classification and segmentation. To the best of our knowledge, there was not a report of sensible failure in the base network. Our proposed attention mechanism was added to this base model, and we did not see any sensible failure case. Understanding the failure case is important, especially in generative models, such as generative adversarial networks (GANs), where two networks compete [46]. If the proposed method is used as a generator of a GAN in future work, then the failure cases need to be analysed.

Adding the proposed attention method to a base method improved its performance compared to the base method. The proposed method was compared with other state-of-the-art (SotA) methods. While the proposed framework does not significantly outperform all existing SotA methodologies, it does achieve a competitive result, matching and surpassing many established frameworks; this shows that the proposed method is valid and contributes meaningfully to the field. It shows clear improvement over the base method, indicating that the attention mechanism does enhance performance. It is designed to be easily integrated into existing point cloud CNNs, allows easy adoption in various architectures, and works directly on the point cloud.

The proposed attention mechanism uses max and average pooling operations, and the pooling operations enhance feature aggregation. The max pooling captures the most prominent features, which helps the network focus on the critical areas of the data. The average pooling reduces noise and improves generalisation, making the proposed approach better than existing ones. With the proposed method, the key aspects of the data are captured.

Whilst the proposed attention model used average pooling and max pooling along the channel axis to extract what the informative and vital inputs are, it did not explore the spatial axis to extract where the key elements are. Hence, it guides the framework to which channel to look in; however, it does not direct the network to where the vital elements are in space. Adopting a spatial attention module to enhance the framework is worth considering in future work. This will help the framework to focus on where in the space is important in the input point cloud features. Additionally, as the proposed attention method is designed to directly work with point cloud data, it can be applied to other different deep neural networks that are working directly with point cloud data.

## Figures and Tables

**Table 1:** The performance of the proposed method when the attention mechanism is added in different positions of the base network, which is compared with the base method, i.e., ConvPoint [9]. The training was performed for 10 epochs for all the methods.

Network/Attention	Number of Epochs	mIoU Score
Base method	10	80.3%
$({Pts}_{6}$$,\;X_{6}$)	10	80.5%
$({Pts}_{5}$$,\;X_{5}$)	10	80.4%
$({Pts}_{4}$$,\;X_{4}$)	10	80.39%
$\left({Pts}_{6},X_{6}\right)\;and\;\left({Pts}_{5},X_{5}\right)$	10	81.45%
$({Ptse}_{5}$$,\;{Xe}_{5}$)	10	80.30%

**Table 2:** Comparison of the proposed method with the base method when they are trained for 200 epochs. The attention mechanism was applied to (${Pts}_{6}$, $X_{6}$) and (${Pts}_{5}$, $X_{5}$).

Network/Attention	Number of Epochs	mIoU Score
Base method	200	83%
Proposed method	200	84.2%
Proposed method using median pooling	200	84.2%

**Table 4:** Comparison of the number of parameters of the proposed method with KPConv [42], the method that achieves the highest accuracy.

Network	Number of Parameters
PointCloud-At	1.3 M
KPConv [42]	14.2 M

## Data Availability

The results of original contributions presented in the study are included in the article, further inquiries can be directed to the authors.
